# Fatal Necrotizing Fasciitis Caused by Pasteurella multocida Without Cutaneous Findings: When Pain Is the Clue

**DOI:** 10.7759/cureus.102296

**Published:** 2026-01-26

**Authors:** David J Vogt, Vanessa Frey, Ruth Valek, Roberto Buonomano

**Affiliations:** 1 Internal Medicine, Spital Limmattal, Schlieren, CHE; 2 Intensive Care Unit, Spital Limmattal, Schlieren, CHE; 3 Infectious Disease, Spital Limmattal, Schlieren, CHE

**Keywords:** necrotizing fasciitis (nf), pasteurella multocida, potential pitfall for misdiagnosis, severe sepsis, soft-tissue infections, zoonosis

## Abstract

We report a case of fatal necrotizing fasciitis in an elderly woman presenting with severe right upper-arm pain and elevated inflammatory markers in the absence of apparent skin changes or diagnostic imaging findings. Despite broad-spectrum antimicrobial therapy and supportive care, the patient deteriorated rapidly and died within 24 hours of hospital admission.

Post-mortem blood cultures identified *Pasteurella multocida*, a zoonotic pathogen commonly found in cats and dogs. Retrospective review of the initial CT scan revealed fascial edema and soft tissue stranding, compatible with necrotizing fasciitis. Necrotizing fasciitis caused by *P. multocida* is exceedingly rare, particularly in the absence of skin disruption or penetrating injury. Close contact with household animals can enable non-bite transmission.

This case highlights an important diagnostic pitfall: severe, localized pain out of proportion to physical findings may be the only early clinical sign of necrotizing fasciitis. Clinicians should remain vigilant for deep soft-tissue infection in septic patients presenting with disproportionate pain, as early recognition, interdisciplinary management, and prompt surgical intervention are essential to improve outcomes.

## Introduction

Necrotizing fasciitis is a rapidly progressive, life-threatening soft-tissue infection characterized by widespread fascial necrosis and high mortality if diagnosis and surgical intervention are delayed [1,2]. Early clinical features are often subtle, and classical cutaneous findings such as erythema, bullae, ecchymosis, or crepitus may be absent in the initial stages, especially in elderly or immunocompromised patients [1,3]. 

*Pasteurella multocida* is a small, non-motile, Gram-negative, facultatively anaerobic rod that forms part of the normal oral flora of cats and dogs [4]. A major virulence factor is its lipopolysaccharide capsule, which facilitates evasion of phagocytosis by immune cells. The pathogen causes respiratory and gastrointestinal diseases in various animal species. In humans, its pathogenicity is generally low, but zoonotic infections are possible. Transmission typically occurs via animal bites or scratches, most often from cats, less commonly from dogs. However, non-bite transmission via licking or close contact has been documented and occurs likely through microabrasions, minor skin breaks or mucosal contact [5,6]. Such exposure may lead to soft tissue infections and rarely respiratory infections. Necrotizing fasciitis caused by *P. multocida* is exceptionally rare. To our knowledge, this presentation has been described fewer than ten times previously in the literature [6-8].

We describe a fatal case of *P. multocida* necrotizing fasciitis in an elderly woman presenting with rapidly progressive sepsis and severe upper-extremity pain but no cutaneous abnormalities and non-diagnostic CT imaging. The case highlights the diagnostic challenge of necrotizing fasciitis when skin changes are absent and image findings are inconclusive.

## Case presentation

Investigations

An 83-year-old female patient presented to the emergency department with a one-week history of pain in the right upper arm and right thoracic region, accompanied by progressive functional decline. On the day of admission, she was no longer able to rise independently from bed. She lived alone with three cats. Her medical history included well-controlled type 2 diabetes mellitus, hypothyroidism, and atrial fibrillation. She was previously fully independent in activities of daily living.

On examination, she was alert and afebrile. Her vital signs showed tachycardia at 146 beats per minute, hypotension with a blood pressure of 81/46 mmHg, and an oxygen saturation of 91% on room air. She had lower extremity edema. Pain out of proportion was elicited on palpation of the right shoulder, upper arm, and right thorax, but there was no visible erythema, swelling, skin discoloration, bullae, or crepitus.

Laboratory tests showed leukopenia (2.8 G/L; reference 4-9.8 G/L), thrombocytopenia (123 G/L; reference 150-400 G/L), markedly elevated C-reactive protein (205 mg/L; reference < 5 mg/L), acute kidney injury with elevated creatinine (253 µmol/L; reference 44-80 µmol/L), hypoalbuminemia (12 g/L; reference 35-52 g/L), elevated lactate (5.3 mmol/L; reference 0.5-1.6 mmol/L), and metabolic acidosis with a base excess of −6.9 mmol/L (reference −3 to 2 mmol/L). Urinalysis revealed leukocyturia, hematuria, and nitrite positivity, suggesting a possible urinary tract infection, in line with her report of pollakiuria. Blood cultures were obtained before antibiotic administration.

A CT scan of the thorax, cervical spine, and right shoulder showed degenerative musculoskeletal changes. No radiologic features were considered diagnostic of acute deep soft tissue infection at the time.

Diagnosis

Given fulfillment of the Sequential Organ Failure Assessment (SOFA) criteria (5 points), a diagnosis of sepsis of unknown origin was made [9]. Due to abnormal urinalysis and pollakiuria, urinary tract infection was considered the most likely infectious source. The patient’s pain was attributed to degenerative changes of the shoulder and cervical spine reported on imaging. Deep soft tissue infection was initially considered but deemed unlikely due to the absence of radiographic or cutaneous findings as well as the presence of an alternative plausible source of infection.

Treatment

Empiric intravenous amoxicillin-clavulanate was initiated to provide broad coverage for common community-acquired pathogens, in line with current local practice recommendations for sepsis of unknown origin. Therapy was soon escalated to piperacillin-tazobactam because of rapid progression to septic shock.

She was transferred to the intensive care unit for management of refractory hypotension requiring vasopressor support (norepinephrine 50 μg/minute, vasopressin 0.04 μg/minute). She developed septic cardiomyopathy, acute hypoxemic respiratory failure, and anuric kidney injury, requiring inotropic support (dobutamine 250 mg/hour), mechanical ventilation, and continuous hemofiltration.

Follow-up and outcomes

The patient’s hemodynamic status deteriorated further, with worsening lactic acidosis, persistent hypotension, progression to anuric renal failure, and declining level of consciousness. She developed circulatory collapse and died 17 hours after initial hospital presentation.

**Figure 1:**
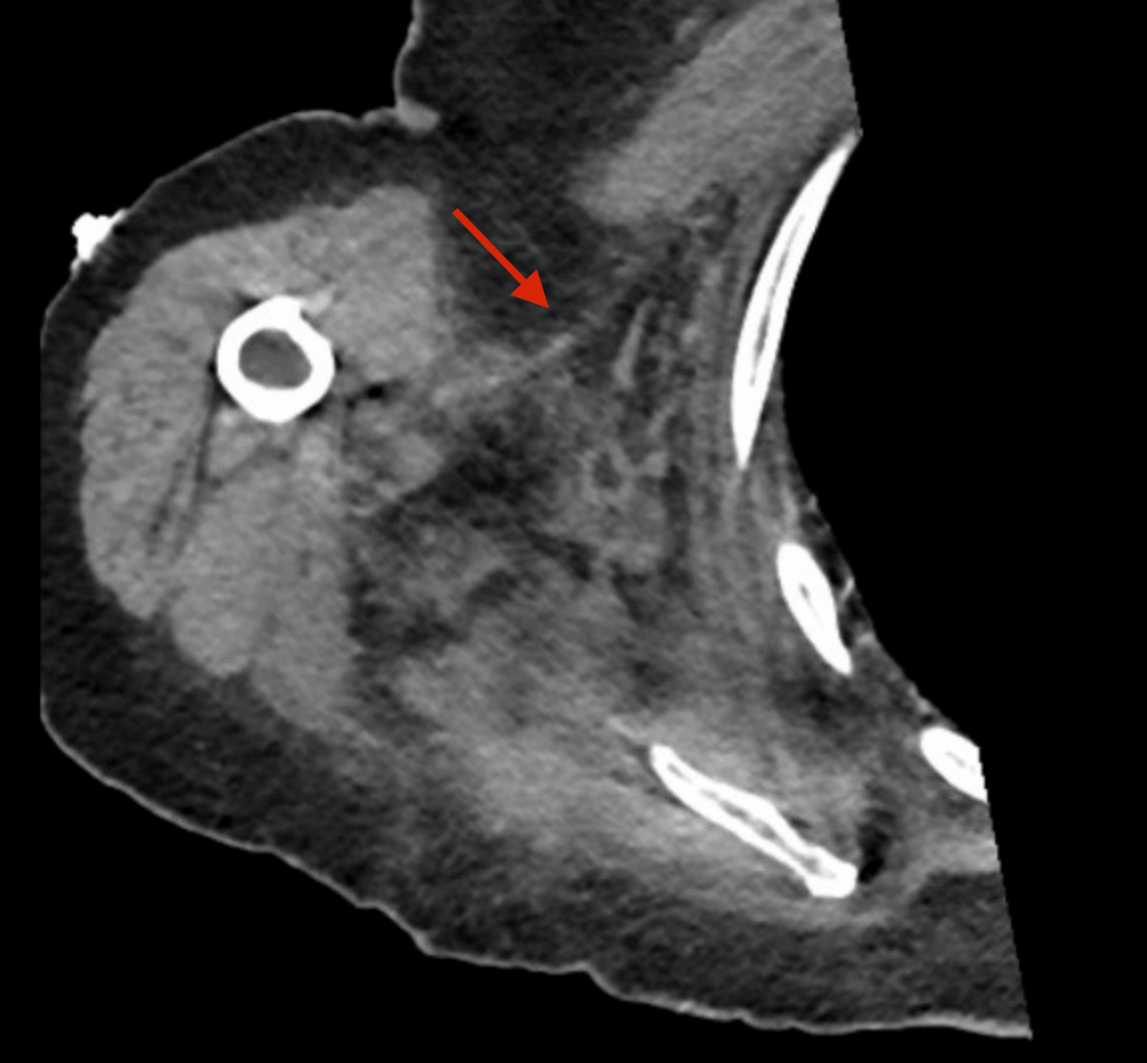
CT scan of the right shoulder and thorax showing right thoracic soft tissue edema (arrow).

Following identification of *P. multocida* in all four post-mortem blood cultures, the initial CT images were retrospectively reviewed in light of the patient’s clinical course. Fascial edema and soft tissue stranding were noted, which, when interpreted alongside the blood culture results and the patient’s rapid deterioration, were consistent with necrotizing fasciitis (Figure 1).

## Discussion

Necrotizing fasciitis is a rare but life-threatening infection of the fascia and subcutaneous tissues, with an incidence of 0.4-15 per 100,000 population depending on region [1,10]. It is most commonly polymicrobial (type I), involving both anaerobic and aerobic bacteria, or monomicrobial (type II), usually due to streptococci and less commonly staphylococci. Sepsis develops as a consequence of both uncontrolled bacterial proliferation and an exaggerated host inflammatory response [11]. Bacteria gain entry through breaches in the skin and may extend into the fascia [1]. Because the fascia is poorly vascularized, bacterial proliferation can occur largely unchecked along the fascial planes, allowing rapid spread of infection. The bacteria may produce potent exotoxins and enzymes such as lipases, proteases, hyaluronidase, and superantigens. These factors additionally facilitate rapid tissue destruction, bacterial spread, and vascular thrombosis, leading to tissue ischemia and necrosis.

Bacterial toxins and pathogen-associated molecular patterns (PAMPs) enter the systemic circulation and trigger a massive cytokine release, endothelial dysfunction, capillary leak, and dysregulated coagulation. This results in systemic inflammatory response syndrome (SIRS) with hypotension, microcirculatory failure, and ultimately septic shock with multiorgan dysfunction. The disease progresses rapidly to extensive tissue necrosis, overwhelming sepsis, and death, with reported overall mortality rates of 14%-34% [3,10,12].

Rapid diagnosis and interdisciplinary treatment, including early antibiotic administration and, often repeated, extensive surgical debridement, are key elements of successful treatment [13]. According to international guidelines for empiric antimicrobial treatment of necrotizing fasciitis, piperacillin-tazobactam in combination with clindamycin should be administered to cover Gram-positive, Gram-negative, and anaerobic pathogens. Clindamycin is added supportively because it inhibits bacterial toxin production, penetrates well into deep tissues, and, unlike beta-lactams, acts independently of the bacterial growth phase, helping to decrease overall bacterial load.

Beyond antimicrobial therapy, surgical management remains the cornerstone of treatment in necrotizing fasciitis. Prompt surgical debridement is pivotal, as delays are associated with increased mortality [14]. Adequate recognition of the extent of infection and performance of sufficiently extensive debridement are challenging and rely largely on the surgeon’s experience. Surgical intervention is essential because antibiotic penetration into the fascia is limited by poor vascularization. Once necrotic tissue develops, it serves as a persistent reservoir of bacteria and toxins, impairs antibiotic penetration, and sustains ongoing infection.

Necrotizing fasciitis remains one of the most challenging soft-tissue infections to diagnose early, particularly in older adults who may present with vague symptoms and blunted inflammatory responses. The diagnostic difficulty stems from the discrepancy between the often subtle external findings and the severe underlying infection. A key clinical red flag is severe pain out of proportion to external findings [1-3]. Although erythema, bullae, ecchymosis, or crepitus are classic clinical features, they may be absent, particularly during the initial phase of disease. In fact, 70%-80% of patients present with pain with nonspecific or no cutaneous findings [15]. Delays in diagnosis are therefore very common, and early recognition is frequently missed [16]. Similar to our case, early laboratory parameters (leukopenia, elevated lactate, and hypoalbuminemia) as well as clinical signs (hypotension, tachycardia, functional decline) typical of a fulminant infectious process are usually present in necrotizing fasciitis. These findings may aid in identifying a patient’s severity of disease in infections and might be used as an early warning sign.

Diagnosis relies heavily on clinical evaluation, since no imaging modality can reliably exclude necrotizing fasciitis. Normal imaging findings should not delay bedside evaluation or surgical consultation. Nevertheless, imaging can play a supportive role in the evaluation of soft tissue infections. Fascial gas collection is the hallmark of necrotizing fasciitis in CT imaging. While this finding is highly specific (>90%), it is often absent early in the course of the disease, limiting its sensitivity (<50%) [17]. Other findings may include inflammatory fat stranding or fascial thickening [18]. Deep fascial thickening and signal alterations (especially T2 hyperintensity) are commonly present in MRI (80-90% sensitivity). However, these changes can be difficult to distinguish from non-necrotizing soft-tissue infections (60-70% specificity), and MRI use in emergency settings may be limited by scan time, availability, and patient stability.

*P. multocida* is a zoonotic pathogen colonizing the oral cavities of domestic cats and dogs [1]. Although typically associated with bite-related soft-tissue infections, non-bite transmission via licking or close exposure is well documented [6,7]. *P. multocida* is usually associated with mild soft tissue infections, but occasionally, more severe courses occur. Other manifestations include respiratory infections and, in rare cases, meningitis, bacteremia, and endocarditis [19,20]. The antibiotic of choice for empiric therapy is amoxicillin/clavulanic acid; resistance is barely reported [21,22]. Third-generation cephalosporins may also be effective.

Necrotizing fasciitis due to *P. multocida* is exceptionally rare, with very few cases reported in the literature [6-8,23-25]. In most of these cases, patients had severe comorbidities (e.g., liver failure) or were immunosuppressed, and presented with skin changes like erythema or bullae. Survival has been reported only in two cases, whereas four cases were fatal.

Typical risk factors for severe invasive infections include advanced age, immunosuppression, and comorbidities [26]. In this unique case, our patient was elderly and had diabetes mellitus, which is a very common comorbidity associated with necrotizing fasciitis [13]. Reflecting the rapid progression in necrotizing fasciitis, the course in this case was fulminant. The absence of visible skin abnormalities and radiographic findings contributed to delayed diagnosis in our patient, despite the presence of pain out of proportion as an early warning sign. In retrospect, the abnormal urinalysis was likely incidental and shows how urinary findings can confound early source identification in septic patients. Early surgical consultation and bedside exploration may have altered management, which underscores the importance of clinical judgement in diagnosing necrotizing fasciitis.

A limitation of this case is the absence of ante-mortem tissue confirmation and the lack of an autopsy. Nevertheless, the combination of microbiologic evidence, clinical deterioration, and retrospective radiologic findings strongly supports necrotizing fasciitis as the cause of fulminant *P. multocida* sepsis, and compensates for the lack of histopathologic confirmation. Given that *P. multocida* typically causes soft-tissue infections, and with CT confirming soft-tissue involvement, other infectious foci are very unlikely. Additionally, the absence of visible skin findings and the rapid clinical trajectory point away from other soft tissue infections like erysipelas or phlegmones, which typically show extensive skin abnormalities in rapidly progressing sepsis.

## Conclusions

This case underscores the importance of maintaining suspicion for necrotizing fasciitis in septic patients who present with pain out of proportion to clinical findings. Early imaging is frequently inconclusive, and diagnosis must rely primarily on clinical assessment, even in the absence of an obvious skin entry site, particularly in elderly or immunocompromised patients. Prompt surgical intervention is essential, as necrotizing fasciitis can progress rapidly without timely debridement.

Finally, this case reinforces that a history of close contact with household animals should prompt consideration of zoonotic pathogens such as *P. multocida*, which, although rare, can cause rapidly progressive and life-threatening infections.
